# Lifestyle habits of adults during the COVID-19 pandemic lockdown in Cyprus: evidence from a cross-sectional study

**DOI:** 10.1186/s12889-021-10863-0

**Published:** 2021-04-23

**Authors:** Ourania Kolokotroni, Maria C. Mosquera, Annalisa Quattrocchi, Alexandros Heraclides, Christiana Demetriou, Elena Philippou

**Affiliations:** 1 Department of Primary Care and Population Health, Medical School, University of Nicosia, 21 Ilia Papakyriakou, 2414, Engomi, P.O. Box 24005, CY-1700 Nicosia, Cyprus; 2 Department of Life and Health Sciences, School of Sciences and Engineering, University of Nicosia, Nicosia, Cyprus; 3 Department of Nutritional Sciences, King’s College London, London, UK

**Keywords:** Lifestyle, Coronavirus pandemic, COVID-19, Lockdown, Mediterranean diet, Physical activity, Stress, Sleep, Social support, Addictions

## Abstract

**Background:**

The COVID-19 pandemic and the widespread adoption of virus control measures have inevitably disrupted efforts to address lifestyle risk factors for non-communicable diseases (NCD). This study aimed to explore the effects of COVID-19 lockdown on all lifestyle medicine pillars, namely diet, physical activity, sleep, stress, social support and use of risky substances.

**Methods:**

This was a cross-sectional study on a convenient sample of adults who resided in Cyprus during the Spring 2020 lockdown. Participants completed an anonymous online questionnaire comprised of six validated tools regarding the following lifestyle behaviours before and during lockdown: adherence to the Mediterranean diet, physical activity, stress and social support levels, sleep pattern and use of risky substances such as smoking and alcohol. Paired before and during lockdown comparisons for each lifestyle pillar were undertaken using Wilcoxon Signed-Rank test and Bowker symmetry Test where response was numerical (non-parametric data) and categorical respectively. Furthermore, stratified analyses for sociodemographic characteristics were performed.

**Results:**

Out of 745 participants, 74% were female and median age was 39 years. Overall participants reported significantly higher perceived stress score (22 v 25, *p* <  0.01), lower social support score (71 v 68, *p* <  0.001), and worse sleep quality score (4 v 5, *p* <  0.01) during lockdown. Mediterranean diet (MD) adherence was moderate and increased significantly only in those practicing religious fasting (score of 6 v 7, *p* <  0.01). Total minutes spent sitting increased (120 v 180, *p* <  0.01) although overall physical activity score did not significantly change. Smoking intensity increased during lockdown whilst frequency of alcohol consumption decreased (p_trend_ = 0.03 and <  0.01, respectively).

**Conclusion:**

Various lifestyle factors were adversely affected by the COVID-19 lockdown in Cyprus. Evidence from this study supports development of holistic lifestyle interventions during and following the pandemic to reduce short and long-term NCD risks by building on lifestyle behaviour strengths and addressing longstanding and emerging gaps and needs.

**Supplementary Information:**

The online version contains supplementary material available at 10.1186/s12889-021-10863-0.

## Introduction

Despite the fact that the world is currently immersed in a global coronavirus (COVID-19) pandemic [1], it is of importance that, globally, non-communicable diseases (NCDs), such as cardiovascular diseases, cancer, and diabetes, remain notable causes of morbidity and mortality [2]. Lifestyle modifiable behaviours, such as unhealthy diets, physical inactivity, tobacco use, and harmful use of alcohol, pose significant risk factors for NCDs [3]. Concurrently, lifestyle factors such as smoking, alcohol use, physical inactivity and obesity, have been identified as risk factors for adverse COVID-19 outcomes [4–7].

Globally, there is concern that the current COVID-19 pandemic has disrupted progress in addressing lifestyle factors to decrease morbidity and mortality [2]. Widespread measures to combat the current COVID-19 pandemic that encourage or require social distancing, self-isolation, in-home lockdown, and/or quarantine undermine attempts towards a healthy lifestyle and pose a mental health threat [8]. A growing number of studies have investigated individual lifestyle habits such as diet, physical activity, stress, sleep and addictions during the first phase of the pandemic and confirmed that in many cases lockdown and other important measures to limit the spread of virus have adversely affected lifestyle habits [9–11]. For example, lockdown measures limited exercise opportunities, reduced physical activity levels, [9, 10, 12], increased food consumption, affected diet quality [11, 13], and impacted sleep [14]. Furthermore, quarantine has been associated with negative psychological effects, including post-traumatic stress symptoms, confusion, and anger with multiple stressors identified, such as longer lockdown duration, infection fears, frustration, boredom, inadequate supplies, inadequate information, stigma, and financial loss [15]. Not surprisingly, during these unprecedented experiences being faced globally due to the COVID-19 pandemic, the wellbeing of people, in the form of stress, anxiety and sleep disturbances, was also affected [16–18].

A smaller number of studies attempted to assess changes in a combination of lifestyle habits or investigate correlations between them [19, 20]. For example, a study in 1254 adults in Spain evaluated lifestyle across 7 domains using the Short Multidimensional Inventory Lifestyle Evaluation tool developed specifically for the lockdown and showed that healthier habits were associated with higher social support, stress management and higher outdoor exposures [19]. Similarly, another study in the Spanish population showed that several health-related behaviors were adversely affected in the first week of lockdown but improved with longer confinement [20]. However, more studies are needed on a comprehensive assessment across a range of lifestyle habits as globally, various measures, some prolonged, targeting movement restriction, such as lockdown, are still in use.

The field of lifestyle medicine is well equipped to address lifestyle factors, as it aims to utilize an evidence-based approach to prevent, treat and even reverse diseases by encouraging healthy behaviours across the six pillars of lifestyle: healthy eating, physical activity, restful sleep, stress management, avoidance of risky substances such as alcohol and smoking, and healthy relationships [21]. This comprehensive approach of combining healthy lifestyle behaviours is known to be associated with increased disease-free life-years [3] and decreased mortality [22]. Thus, assessing needs based on this approach will be important for the design of lifestyle interventions during this period.

Thus, our study aimed to investigate lifestyle changes across all lifestyle pillars in adults in Cyprus during the first phase of the COVID-19 pandemic, where a lockdown was implemented between 15th March and 21st May 2020. This study aims to inform relevant stakeholders on the well-being priority needs of a population which practices social distancing or is in lockdown thereby aiding the design of holistic lifestyle interventions targeting multiple health behaviours both during and after social distancing to reduce the risk of chronic disease in the short and long term.

## Methods

### Design and setting

This was a cross-sectional web-based questionnaire survey conducted between 10th April and 12th May 2020, designed and performed in accordance with the Declaration of Helsinki. A convenience sample was recruited through social media and institutional and community social network mailing lists. Study participation was anonymous and informed consent was obtained before study enrollment. Following self-completion of the study questionnaire either in the Greek or English language, participants received digital educational material with practical tips on ways to maintain a healthy lifestyle during lockdown.

The Cyprus lockdown was gradual and started on March 10th 2020 with the closure of schools and universities and the prohibition of gatherings of more than 75 people. This was followed by closure of entertainment areas (e.g., malls, hotels, cinemas) and the application of the “1 person per 8 square meters” measure to all public areas on March 15th 2020. On March 24th, the majority of retail stores closed. The country went into a strict lockdown between 31st March and May 3rd when airports were closed (repatriation allowed and individuals arriving from abroad were isolated in quarantine hotels for 14 days), a night curfew was implemented and an “once a day” allowance of going out of the house was given for essential movements. Gatherings were prohibited and intercity movements were only allowed for essential work. Between May 3rd and May 21st, daily allowance to go out of the house increased to maximum of 3 times per day. After May 21st, there was a gradual lifting of restriction measures [23].

### Study population

The study population consisted of adults ≥18 years who were living in Cyprus during the period of the Spring 2020 lockdown.

### Assessment tools

The study questionnaire consisted of six widely used tools validated in both English and Greek in order to assess the six pillars of lifestyle medicine: nutrition, physical activity, sleep, stress, social connection, and risky substance use (alcohol). It also included questions on socio-demographic characteristics of participants, including self-reported height and weight, and questions assessing tobacco use. Participants were asked to provide their responses concerning: (a) February 2020, the month preceding the emergence of coronavirus in Cyprus and (b) the period in lockdown.

The validated tools used were the: (a) Mediterranean Diet Adherence Screener (MEDAS) [24], (b) International Physical Activity Questionnaire (IPAQ) [25], (c) the Pittsburgh Sleep Quality Index (PSQI) [26], (d) Perceived Stress Scale-14 (PSS-14) [27], (e) the Medical Outcomes Study – Social Support Survey (MOS-SSS) [28], and (f) Alcohol Use Disorders Identification Test (AUDIT-C) [29].

### Statistical analysis

Scores were calculated in line with published tool-specific scoring instructions. Descriptive analyses were performed to calculate absolute and relative frequencies for categorical variables and median and interquartile ranges (IQR) for numerical variables. To evaluate the effects of lockdown on socioeconomic, anthropometric and lifestyle questions, paired before (referring to month of February) and after (referring to the time during lockdown) comparison was undertaken. Wilcoxon Signed-Rank test and Bowker symmetry Test were performed for questions where response was numerical and categorical respectively. Non parametric tests were used as numerical variables were checked and were not found to follow normal distribution. Participants with missing values in an outcome variable were excluded from any analysis on that variable.

Paired before and during lockdown comparisons for MD adherence, IPAQ, PSQI, MOS-SSS PSS-14 and AUDIT-C scores were performed for the overall cohort, as well as stratified by independent sociodemographic variables, such as age group, gender, nationality, education level, marital status, number of people living in the household, residence in urban/rural areas, employment status, change in working conditions, baseline household monthly net income and change in monthly income. Lastly, before and during lockdown differences in MD adherence, IPAQ, PSQI, MOS-SSS, PSS-14 and AUDIT-C scores were correlated between them, while adjusting for age and gender (and fasting status for MD adherence), using partial Spearman’s rank correlation. A *p*-value < 0.05 was regarded as significant in all analyses. Statistical analyses were performed using STATA® v.16 (StataCorp., USA) and R statistical software packages.

## Results

### Participant characteristics

The socio-demographic characteristics of the 745 participants are presented in Table 1. Briefly, the median age was 39 years (IQR: 13 years); 73.8% were female. The great majority lived in urban areas (85.0%) and had attained university education at undergraduate (40.0%) or postgraduate level (50.3%). Two thirds of participants were employed (66.3%) and married, living with a partner or in a partner relationship (61.2%). Almost half (46.9%) had a baseline household net-income < 2000 euro per month. During the lockdown, working conditions changed for three in four participants (74.4%). Among them, 39.1% worked more hours, 36.7% worked less hours and 24.3% suspended work. Among those working, 63.8% worked from home, 9.4% went to the workplace, and 18.5% did both. Overall, 74.2% did not report any diagnosed health conditions.

**Table 1:** Socio demographic and health related characteristics

Socio-demographic characteristics		Total (***n*** = 745)^**a**^	
		N	%
Gender	Female	550	73.8
	Male	195	26.2
Age	Median (IQR)	39 (13)	
Age groups	18–29	218	29.3
	30–39	162	21.7
	40–49	174	23.4
	50–76	191	25.6
Nationality	Cypriot	603	81.3
	Greek	75	10.1
	European	41	5.5
	Other	23	3.1
Area	Urban area (city)	633	85.0
	Rural area (village)	112	15.0
Educational attainment	Primary (Primary school)	2	0.3
	Secondary (High School)	70	9.4
	Tertiary (University / College at undergraduate level)	298	40.0
	Tertiary (University / College postgraduate or doctoral level)	375	50.3
Employment status	Student	155	20.8
	Unemployed for the whole year	22	3.0
	Unemployed for part of the year	20	2.7
	Employed	494	66.3
	Retired	54	7.2
Marital status	Single	232	31.1
	Married / Living together / Relationship	456	61.2
	Divorced	47	6.3
	Widowed	10	1.3
N. of people in household	Median (IQR)	3 (2)	
Income in euro (in the Month before Lockdown)	< 1000	101	15.7
	1000–2000	200	31.2
	2000–3000	175	27.3
	3000–4000	92	14.3
	> 4000	74	11.5
Have your working conditions changed during the month in lockdown?	No	95	12.8
	Yes	552	74.4
	Not Applicable	96	12.9
If yes, how have your working conditions changed during the month in lockdown?	Working more hours	211	39.1
	Working less hours	198	36.7
	Not working	131	24.3
If working different hours, has your place of work changed during the month in lockdown?	Working from home only	293	63.8
	Working outside of home only	43	9.4
	Working both from home and outside of home	85	18.5
**Health related characteristics**			
BMI in month before lockdown	Median (IQR)	24.0 (6.0)	
BMI in lockdown	Median (IQR)	24.2 (6.2)	
Were you fasting during the month of lockdown?	No	559	75.0
	Yes	186	25.0
Do you currently have any diagnosed health conditions?	No	553	74.2
	Yes	192	25.8

^a^Missing values: nationality (3), income in euros (103), Have your working conditions changed during the month in lockdown? (2), If yes, how have your working conditions changed during the month in lockdown? (12), If yes, has your place of work changed during the month in lockdown? (26), BMI in month before lockdown (3), BMI in lockdown (2)

### Comparison of lifestyle habits before and during lockdown

In Table 2, the lifestyle habits of participants (Diet, Physical Activity, Stress, Sleep, Social Connection and Use of Risky Substances) are compared before and during lockdown. Most lifestyle habits were adversely affected during lockdown, as indicated by changes in the overall median questionnaire scores and the shift in score distribution towards worsening values (Supplementary Figure 1). Changes in scores within individual areas of each lifestyle pillar are presented in more detail in Supplementary Tables 1, 2, 3, 4 and 5.

**Table 2:** Lifestyle habits (Diet, Physical activity, Stress, Sleep, Social connection, Use of risky substances) before and during lockdown

Lifestyle habits	The Month before Lockdown	Lockdown	Change in score^b^ (% of participants)		*P* value ^c^
	Median (IQR) ^a^		Decrease	Increase	
**Diet**					
Mediterranean Diet Score (range: 0–14)	6 (2)	6 (3)	22.7	31.9	< 0.01
**Physical Activity**					
MET	792 (1880)	813 (1815)	37.0	43.8	0.16
Time spent sitting (mins)	120 (340)	180 (466)	14.5	67.1	< 0.01
Physical Activity Score (%)					
Low	49.4%	48.9%	21.2	21.5	0.95
Moderate	25.2%	26.3%			
High	25.4%	24.8%			
**Stress**					
PSS-14 overall score (0–56)	22 (9)	25 (12)	21.7	57.9	< 0.01
**Sleep**					
Global PSQI score (range: 0–21)	4 (4)	5 (4)	25.3	49.1	< 0.01
Sleep latency (0–3)	1 (1)	1 (2)	6.1	40.6	< 0.01
Daytime dysfunction (0–3)	1 (1)	1 (1)	12.3	28.5	< 0.01
Sleep efficiency (0–3)	0 (1)	0 (0)	15.6	14.1	< 0.01
Sleep medication (0–3)	0 (0)	0 (0)	1.2	4.3	< 0.01
Sleep quality (0–3)	1 (1)	1 (2)	10.1	26.8	< 0.01
**Social Support**					
Overall Support Index (0–100)	71.1 (31.58)	68.4 (36.8)	43.6	18.0	< 0.01
Emotional/informational support	71.9 (37.5)	65.6 (40.6)	28.9	12.5	< 0.01
Tangible support	75.0 (43.8)	75 (43.75)	20.7	13.4	< 0.01
Affectionate Support	75.0 (50)	75 (50)	24.6	9.7	< 0.01
Positive Social Interaction	75.0 (50)	66.7 (58.3)	34.0	11.0	< 0.01
**Use of Substances**					
Cigarettes, cigars and e-cigarettes (total n)	10 (14)	10 (8)	28.1	43.8	0.03
Alcohol Use Disorders Identification Test (AUDIT-C)	2 (2)	1 (2)	29.4	11.5	< 0.01
Alcohol Use Disorders Identification Test (AUDIT-C) score risk (%)					
No or low risk	77.2%	79.8%	7.04.6	4.67.0	0.06
High risk	22.7%	20.2%			
Frequency of alcohol consumption (%)					
Never	22.3%	36.2%	26.0	11.5	< 0.01
Monthly or less	29.4%	27.2%			
2–4 times a month	33.2%	19.9%			
2–3 times a week	10.3%	8.9%			
4 or more times a week	4.8%	7.8%			
Number of alcoholic drinks on a typical day (%)					
0	53.1%	57.5%	13.1	9.1	0.08
1 or 2	39.3%	36%			
3 or 4	6.0%	4.8%			
5 or 6	1.2%	1.1%			
7,8 or 9	0.3%	0.3%			
10 or more	0.1%	0.3%			
Six or more alcoholic drinks on one occasion (%)					
Never	78.6%	88.3%	11.6	3.1	< 0.01
Less than Monthly	12.2%	6.3%			
Monthly	6.6%	2.9%			
Weekly	2.5%	1.8%			
Daily or Almost Daily	0.1%	0.7%			

^a^Questionnaire Scores for reporting different lifestyle pillar habits are presented as median values (IQR) with the exception of alcohol consumption where the percentage of participants per consumption frequency is presented. The MEDAS score ranges from 0 to 14, with higher scores indicating higher adherence to the Mediterranean Diet. The IPAQ score, categorizes participants into levels of Low, Moderate and High physical activity. The PSQI score, ranging from 0 to 21, assesses sleep quality with higher scores (> 5) representing poor sleep quality. The MOS-SSS ranges from 0 to 100 with higher scores indicating higher levels of social support. The PSS-14 score ranges from 1 to 14, and higher scores are associated with increased perceived stress. The AUDIT-C score ranges from 0 to 12; a score of 4 or more in men and a score of 3 or more in women is indicates elevated risk for hazardous drinking or active alcohol use disorder. Missing values for all responses included in score calculations were in the range of < 5%

^b^Change in score refers to the Month in Lockdown compared to February 2020 (i.e. - the Month before Lockdown)

^c^Signrank test was used for the comparison of median questionnaire scores between February 2020 (i.e. - the Month before Lockdown) and lockdown and Bowker symmetry Test for comparison of categorical variables

MD adherence remained moderate during lockdown (median 6, IQR 3) although one-third of participants reported a higher score (31.9%) (*p* <  0.01). In particular, there was increased consumption of most components of the MD including those healthy (e.g., fruit, vegetables, legumes) and less healthy (e.g., sweet beverages and commercial sweets), whereas preferential consumption of white vs. red meat did not change. Subgroup analyses demonstrated that increased adherence was only significant among participants who started fasting, as per the Greek Orthodox religion, during lockdown. Among these participants (25% of total study population), median MD adherence score increased by 1 unit (*p* <  0.01). Of note, Body Mass Index (BMI) increased slightly but significantly during lockdown (24.2 vs. 24.0, *p* <  0.01).

As expected, increasing sedentary behaviour was reported by most participants during lockdown (180 vs.120 min sitting, *p* <  0.01). However, the overall physical activity score did not significantly change (*p* = 0.95); 60% of participants did not report any change and the remaining 40% were split between increased and decreased activity levels. However, there was a significant increase in the average weekly energy daily expenditure in walking during lockdown (Supplementary Table 2, MET (one Metabolic Equivalent - min/week 297 vs. 231, *p* <  0.01). In contrast, MET-min/week spent in moderate or vigorous physical activity were lower during the lockdown period, albeit non-significant except in younger participants (18–29-year-old, students).

Being in lockdown was also significantly associated with an increase in perceived stress (25 vs. 22, *p* <  0.01). Almost 6 in 10 participants (57.9%) reported higher stress scores during lockdown. Similarly, sleep quality was negatively affected; the median Global PSQI score significantly increased (5 vs. 4, *p* <  0.01) and one in two participants reported worsening scores. Moreover, the proportion of participants with poor sleep quality (global PSQI score > 5) increased during lockdown (40.4% v 26.0%, *p* <  0.01). Regarding the individual PSQI score components (sleep latency, daytime dysfunction, sleep medication and sleep quality), all increased during lockdown (*p* <  0.01), demonstrating a worse sleep experience, except sleep efficiency, which marginally improved during lockdown (*p* <  0.01). Social support was also adversely impacted: the overall support index decreased significantly in lockdown (68.4 vs. 71.1, *p* <  0.01), almost half of the participants (43.6%) reported lower support scores. A significant decrease was observed in all overall support index components, namely emotional/informational support, tangible support, affectionate support and positive social interaction (*p* <  0.01).

Pertaining substance use, 43.8% of smokers increased their smoking intensity during the month in lockdown (*p* <  0.03). In contrast, the median AUDIT-C score decreased significantly in lockdown (2 vs 1, *p* <  0.01) as a consequence of the reduction in the overall frequency but also quantity of alcohol consumption. Indicatively, 26% of the sample decreased consumption frequency compared to 11.5% who increased consumption, while abstinence also increased (36.2% v 22.3%). Regarding quantity of consumption, even though the number of alcoholic drinks per day did not change significantly (*p* = 0.08), more participants reported a decrease rather than an increase in the frequency of binge drinking (≥6 alcoholic drinks on one occasion) during lockdown (11.6% vs. 3.1%, *p* < 0.01). Similarly, the proportion of high-risk drinkers defined as per the AUDIT -C score decreased in lockdown (22.7% vs 20.2%, *p* = 0.06), whilst this decrease was more pronounced in men (29.4% vs 23.3%, *p* = 0.04, data not shown).

Stratified analysis by socio-demographic characteristics (data not shown) showed that sleep, social support and stress scores increased significantly across all socio-economic strata. Physical activity levels decreased in the younger age and student groups. Nonetheless, higher MD adherence was seen in both genders, younger (18–29 y.o) and older age groups (50–76 y.o.), those living in urban areas, and those with higher educational attainment.

### Correlation between differences in lifestyle scores during lockdown

In Table 3, the correlations between before and during lockdown differences in lifestyle scores, adjusted for potential confounders, are shown. Weak to moderate significant associations were observed between perceived stress and sleep quality index, overall support index and perceived stress, and between overall support index and sleep quality index. More specifically, a positive moderate correlation (*r* = 0.4064, *p* < 0.01) between differences in perceived stress and sleep quality index before and during lockdown, indicating that an increase in perceived stress was associated with worsening sleep quality. Overall support index score difference was negatively correlated with perceived stress difference (*r* = − 0.3742, *p* < 0.01) and sleep quality index difference (*r* = − 0.2253, *p* < 0.01), showing that a decrease in the overall support index during lockdown was associated with an increase in perceived stress and worsening sleep quality.

**Table 3:** Association between lifestyle habits during the month of lockdown (^a^)

	Diet score difference ***n*** = 745	PA score difference ***n*** = 745	PSQI score difference ***n*** = 727	MOS-SSS score difference ***n*** = 745	PSS-14 score difference ***n*** = 745
**Diet score difference**	*r* = 1.0000				
**PA score difference**	*r* = 0.0535 *p* = 0.15	*r* = 1.0000			
**PSQI score difference**	*r* = −0.0527 *p* = 0.16	*r* = −0.0911 *p* = 0.01	*r* = 1.0000		
**MOS-SSS score difference**	*r* = 0.0402 *p* = 0.27	*r* = 0.0329 *p* = 0.37	*r* = −0.2253 *p* < 0.01	*r* = 1.0000	
**PSS-14 score difference**	*r* = −0.0627 *p* = 0.09	*r* = −0.0794 *p* = 0.03	*r* = 0.4064 *p* < 0.01	*r* = − 0.3742 *p* < 0.01	*r* = 1.0000

^a^Spearman’s rank Correlation analysis between score differences - All adjusted for Gender and Age, diet also adjusted for Fasting

Lastly, significant but very small negative correlations were observed between differences in physical activity score and sleep quality index (*r* = − 0.0911, *p* = 0.01) and perceived stress (*r* = − 0.0794, *p* = 0.03). A decrease in physical activity scores during lockdown was associated both with worse sleep quality and increased perceived stress. Significant correlations were not observed between diet scores and any other lifestyle pillars.

## Discussion

### Summary of findings

Several studies evaluating lifestyle habits during the COVID-19 pandemic focussed on individual factors such as diet [11], physical activity [10] or psychological health [9, 18]. To the best of our knowledge, our study is one of few that used a comprehensive approach to investigate changes across all six pillars of lifestyle medicine during the COVID-19 pandemic in adults before and during strict lockdown measures. It is also the first study in Cyprus to assess the lifestyle of the local people under these extraordinary circumstances. The study findings show most lifestyle behaviours were adversely affected. Specifically, participants reported being more stressed, having worse sleep quality and lower social support. Furthermore, though participants reported eating more in terms of portions, their quality of diet, however, did not seem to change, with average MD adherence score being moderate before and during lockdown. Overall, physical activity score did not change during lockdown; however, there was an increase in energy expenditure in walking along with an increase in time spent sedentary. A large number of smokers reported increased smoking intensity whereas overall alcohol consumption decreased. A significant correlation was observed between some lifestyle behaviours, with more pronounced effects seen between sleep, stress and social support.

### Main body

Similar to other studies that assessed a range of lifestyle habits during lockdown [19, 20, 30], our study showed consistent findings in regard to the adverse effects and the interlinked association of sleep, stress and social support. For example, the multi-country study by Ammar et al. of 1047 adults across 3 continents showed a positive correlation of mental health with higher social support and a negative correlation with poor sleep [30]. Similarly, the Spanish study of 1254 adults showed that higher social support, stress management and higher outdoor exposures were the most important factors associated with better health behaviours [19]. There was also agreement between our findings and those of the other studies in regard to the higher food consumption and sedentary time during lockdown [19, 20, 30]. However, there was variation in the findings in regards to physical activity changes which could be explained by differences in restriction measures and population baseline physical activity levels [20, 30].

Regarding our findings in each individual pillar and starting with diet during lockdown, participants in our study were affected in various ways. Firstly, diet quality, as reported by the MEDAS score, was moderate. Whilst diet quality seemed to improve in some areas but not in others during lockdown, there was an overall improved MD adherence score amongst participants who were fasting, as per the Greek Orthodox religion tradition. This is not surprising since fasting is a plant-based diet, thus closer to the original MD [31]. Agreeing with our study, adherence to MD during lockdown was moderate in an Italian study with 3533 participants aged 12–86 years. This study reported an increase in the sense of hunger and appetite as well as perceived weight gain in almost half of the participants [32]. Similarly, in another Italian study, half of participants reported higher food consumption as a result of eating more “comfort food” (sweets and salty snacks) but also fruits [11], whereas a study from Poland reported increased snacking between meals especially amongst the obese [33]. The ECLB-COVID19 International Online Survey also showed an increase in the number of meals and snacking during confinement and a higher unhealthy diet score [30]. However, the Spanish COVIDiet study, which also assessed adherence to MD before and after lockdown, adherence to the MD increased significantly from 6.53 +/− 2 to 7.34 +/− 1.93. COVIDiet participants with higher MD adherence decreased intake of sweet/carbonated beverages, red meat and pastries by 16–18%, yet increased fruit and vegetable intake by around 12% [34]. Similar to our findings, COVIDiet participants with postgraduate education had higher MD adherence.

Pertaining exercise, the average weekly activity score per participant in our population did not change during lockdown. These findings are in contrast to a recent systematic review on physical activity and sedentary behaviour during COVID19 lockdown in healthy adults which included 44 studies that in their majority did not use a validated PA measurement tool and suggested a reduction in physical activity levels of individuals in lockdown in most countries [12]. For example, an online survey of 1471 adults in Australia reported a negative change in the physical activity of almost half of the participants [9] whereas a study in Italy showed a significant decrease in the weekly MET-min score across all activity categories in 2524 adults [35]. However, just over 75% of the Italian participants had moderate or high physical activity levels before lockdown and the negative impact of lockdown was mostly seen in these individuals Similarly, the systematic review also showed that people with higher pre-lockdown physical levels were more likely to have a larger decrease in PA levels during lockdown [12]. This is in agreement with our findings that showed a decrease in physical activity levels only in those with moderate and vigorous pre-lockdown physical activity levels. In our study however, half of our participants had low physical activity levels. Individuals classified as low active before lockdown showed a significant increase in their physical activity levels in lockdown as demonstrated by the study of Rodrigo et al. in 1155 adults in Spain [13]. This can explain the fact that in our population, the number of participants who spent time walking increased during lockdown Walking is usually a preferred exercise amongst less active individuals [36]. Moreover, in Cyprus during spring, walking was likely a well-suited outdoor activity for families and seniors. This increase in energy expenditure in walking however was negated by the decrease in moderate and vigorous activity, thus explaining the overall picture of no change in physical activity in our population. Finally, and unsurprisingly, staying at home with a “once a day” allowance to go out led to an increase in the time participants spent sitting and in other sedentary activities, something evidenced by other studies [9, 35] and a recent systematic review on physical activity and sedentary behaviour during COVID-19 lockdown [12].

Sleep, stress, and social support are important interrelated factors in lifestyle medicine [21]. During the COVID-19 lockdown, significant associations were reported between them in studies that evaluated stress and sleep [16] and social support and stress [37]. To our knowledge, few other studies to-date have evaluated social support, sleep, and stress [19, 38]. A smaller study (*n* = 170) in China evaluated persons under self-isolation [38] and showed that low levels of social capital were associated with increased stress, which in turn reduced sleep quality.

In our study, social support decreased during COVID-19 lockdown, which differs from results seen in studies in the US [39] and Egypt [37], where social support increased. This difference may be driven by factors such as timing of the study and degree of lockdown measures as well as societal and cultural differences. Decreased social support in our study was associated with increased perceived stress (*r* = − 0.3742, *p* < 0.01), related to findings of other studies showing the adverse effects of loneliness and lack of social support on stress and mental health during the COVID-19 pandemic [39–41]. Additionally, our study confirms other findings during the COVID-19 pandemic that higher perceived stress is associated with lower sleep quality [16] and that the proportion of those with poor sleep quality increased [42]. A recent systematic review and meta-analysis reported a global pooled prevalence rate of sleep problems of 35% [14]. In our population and although global sleep quality significantly changed in our participants during lockdown (global PSQI score: 4 before vs. 5 during lockdown, *p* < 0.01), it is noteworthy that both before and during lockdown our respondents, overall, had “good” sleep quality (global PSQI score ≤ 5).

Given the association between stress, anxiety and substance use [43], smoking and alcohol consumption frequency and/or intensity during lockdown were expected to increase in some people due to higher stress levels and decrease in others who smoke or drink socially. Findings from our study confirm the above; 43.8% of smokers increased and 28.1% decreased the daily number of cigarettes smoked during the lockdown. Similarly, the overall frequency of alcohol consumption increased in 11.5% and decreased in 26% of participants, while the number of drinks consumed showed a similar pattern.

Regarding smoking, similar findings have been found in a study conducted during the COVID-19 lockdown in the US, where approximately a quarter of participants reduced smoking and a third increased their motivation to quit, whilst 30% increased their smoking [44]. A similar survey conducted across five countries (Italy, India, South Africa, UK, and US) including 6800 smokers under a variety of lockdown measures, found that e-cigarette consumption marginally increased during lockdown [45]. The latter study also revealed that in-home smoking increased in Italy and India among exclusive tobacco cigarette smokers. Both studies note that smoking behaviour of participants was also affected by the perception of increased risk of infection or higher COVID-19 disease severity [44, 45]. Although we did not assess perceptions of infection related to smoking, it is very likely that our participants who reduced or quit smoking during lockdown had similar concerns or that the strict Cyprus lockdown measures prevented social smoking.

Concerning alcohol, there are conflicting findings in the literature. Our study findings are in line with an Italian survey reporting a 36.8% reduction in alcohol intake, probably due to reduced social drinking [11]. Conversely, a study conducted in Poland reported an increase in alcohol consumption in approximately 14% of participants, although more pronounced in alcohol addicts [33]. Similarly, UK evidence on drinking habits during COVID-19 lockdown [46] saw elevation in the proportion of risky drinkers. This is in contrast to our findings, showing a much higher decrease than increase (11.6% vs. 3.1%) in high-risk drinking (≥6 alcoholic drinks on one occasion) during lockdown. Similarly, the ECLB-COVID19 International Online Survey also showed a reduction in binge drinking at a global level [30]. Similar to our findings, in the UK study the proportion of people drinking less during lockdown was similar or exceeded the proportion of those drinking more [46]. Furthermore, a survey conducted by the charity Alcohol Change UK [47] also revealed that one in five participants drunk as a response to stress or anxiety during the lockdown and more than one in three acted to manage their drinking, with 7% stopping altogether. Of note, in our study the proportion of people reporting never drinking increased (36.2% vs. 22.3%).

### Strengths and limitations

Our study had a few strengths and limitations. The study used validated assessment tools to evaluate and compare habits across all lifestyle pillars before and during a strict lockdown in a relatively large group of participants. Despite the use of validated tools, recall bias cannot be excluded due to the self-reporting nature of the assessment tools and the fact that they are not designed to measure health behaviours in the past. Thus, the measurements of the outcomes in the period before lockdown might be less accurate although there is no reason to believe that such bias would have occurred differentially in regard to people’s changes in health behaviours between the two time periods. The convenience sampling method, however, led to overrepresentation of female, well-educated and urban-living participants who were possibly more health conscious. Nonetheless, there was reasonably good representation of all ages (12% of adult population over the age of 65) and income groups (median monthly income in Cyprus in first quadrant of 2020 was 2000 euros) [48]. However, despite our population not being representative of Cyprus, the adverse changes in the lifestyle habits seen during lockdown in our study would have probably been more pronounced in a lower socio-economic, less health-conscious population setting. Health related outcomes and particularly mental health have been more severely impacted by the pandemic in lower socioeconomic groups in a study of 1004 participants living in Vienna [49].

## Conclusions

Our study findings suggest the potential diverse and interlinked effects of the COVID-19 pandemic and the relevant control measures on all six lifestyle medicine pillars. As the COVID-19 pandemic endpoint is not yet known and measures to control the spread of the SARS-CoV-2 virus, such as partial and total lockdowns, will continue, the design of interventions to promote positive lifestyle behaviours is crucial. Such interventions should: (a) support maintenance of “good” lifestyle habits, as in the good sleep quality and increased opportunities for walking seen in our population; (b) address longstanding needs and gaps, such as increasing adherence to the MD; and (c) deal with emerging needs, especially regarding the interlinked triad of stress, sleep and social connection. As the short- and long-term effects of the pandemic on chronic diseases are still unknown, supporting development of holistic lifestyle interventions is of paramount importance.

## Supplementary Information

**Additional file 1: Supplementary Figure 1.** Dot plots showing Month before Lockdown and Month in Lockdown values for Mediterranean Diet Score, Physical Activity Score, PSS-14 score, PSQI score, Overall Support Index, Total number of cigarettes, cigars and e-cigarettes smoked, and AUDIT-C score.

**Additional file 2: Table S1.** Adherence to Mediterranean Diet before and during Lockdown. **Table S2.** Physical Activity levels before and during Lockdown. **Table S3.** Stress levels before and during Lockdown. **Table S4.** Sleep before and during Lockdown. **Table S5.** Social Connections before and during Lockdown.

## Data Availability

The datasets used and/or analysed during the current study are available from the corresponding author on reasonable request.
