# Supplementary material for: Lifestyle habits of adults during the COVID-19 pandemic lockdown in Cyprus: evidence from a cross-sectional study
Source: BMC Public Health. 2021 Apr 23;21:786. doi: 10.1186/s12889-021-10863-0 (PMC8064698; doi:10.1186/s12889-021-10863-0)
Supplement: Supplementary file 1 — Additional file 1: Supplementary Figure 1. Dot plots showing Month before Lockdown and Month in Lockdown values for Mediterranean Diet Score, Physical Activity Score, PSS-14 score, PSQI score, Overall Support Index, Total number of cigarettes, cigars and e-cigarettes smoked, and AUDIT-C score. [file 12889_2021_10863_MOESM1_ESM.docx]

**Title Page**

**Supplementary Figure 1**

**Lifestyle habits of adults during the COVID-19 pandemic lockdown in Cyprus: evidence from a cross-sectional study**

**Authors Information:**

Dr Ourania Kolokotroni, Department of Primary Care and Population Health, Medical School, University of Nicosia *

Dr Maria C Mosquera, Department of Primary Care and Population Health, Medical School, University of Nicosia *

Dr Annalisa Quattrocchi, Department of Primary Care and Population Health, Medical School, University of Nicosia

Dr Alexandros Heraclides, Department of Primary Care and Population Health, Medical School, University of Nicosia

Dr Christiana Demetriou, Department of Primary Care and Population Health, Medical School, University of Nicosia

Dr Elena Philippou, Department of Life and Health Sciences, School of Sciences and Engineering, University of Nicosia. Department of Nutritional Sciences, King’s College London.

*Equal contribution


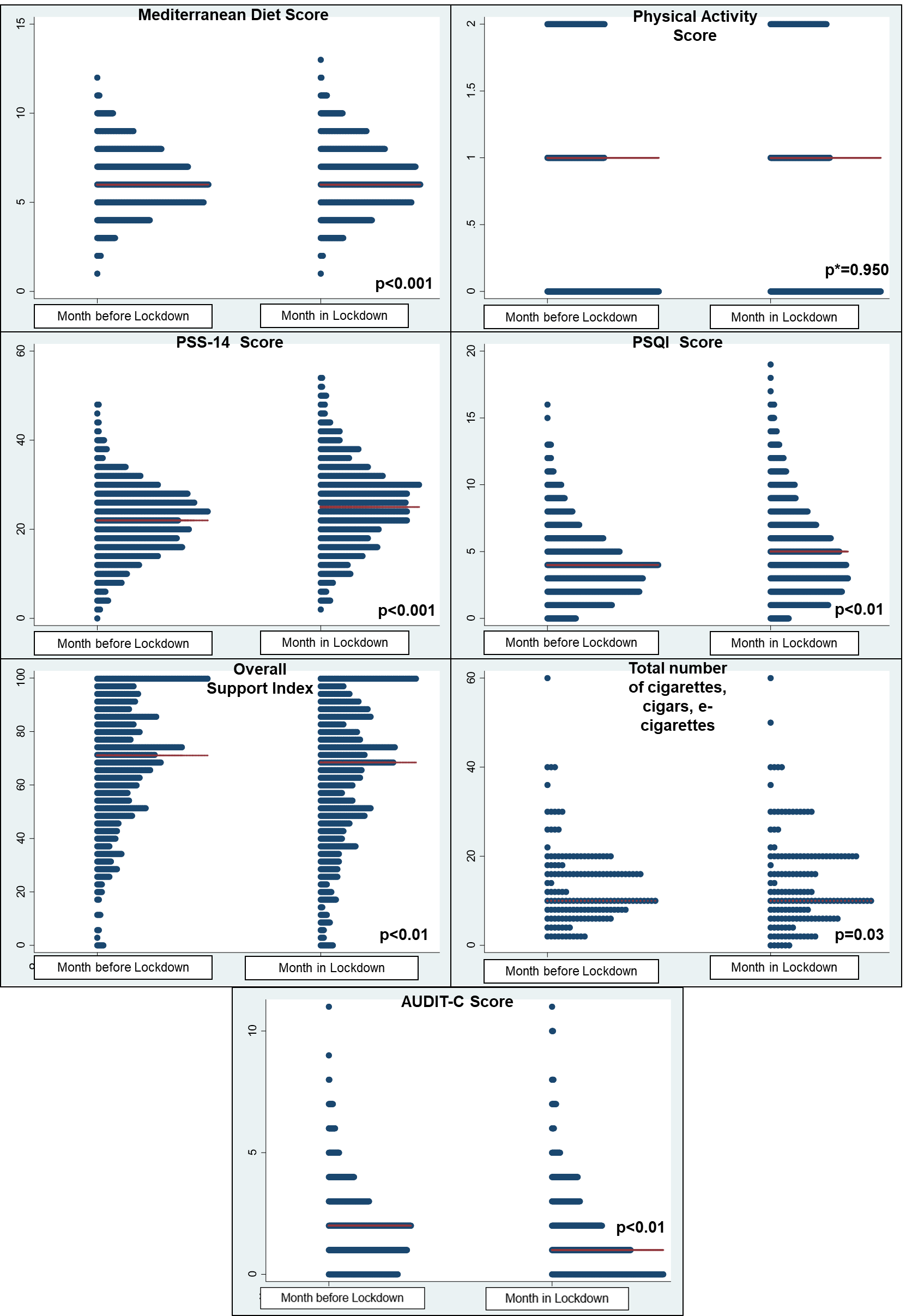


**Supplementary Figure 1** – Dot plots showing Month before Lockdown and Month in Lockdown values for Mediterranean Diet Score, Physical Activity Score, PSS-14 score, PSQI score, Overall Support Index, Total number of cigarettes, cigars and e-cigarettes smoked, and AUDIT-C score.

P-values for all variables are from the Signrank test, with the exception of Physical Activity Score for which the Bower symmetry test was used.
