# Supplementary material for: Lifestyle habits of adults during the COVID-19 pandemic lockdown in Cyprus: evidence from a cross-sectional study
Source: BMC Public Health. 2021 Apr 23;21:786. doi: 10.1186/s12889-021-10863-0 (PMC8064698; doi:10.1186/s12889-021-10863-0)
Supplement: Supplementary file 2 — Additional file 2: Table S1. Adherence to Mediterranean Diet before and during Lockdown. Table S2. Physical Activity levels before and during Lockdown. Table S3. Stress levels before and during Lockdown. Table S4. Sleep before and during Lockdown. Table S5. Social Connections before and during Lockdown. [file 12889_2021_10863_MOESM2_ESM.docx]

**Title Page:**

**Supplementary Tables 1-5**

**Lifestyle habits of adults during the COVID-19 pandemic lockdown in Cyprus: evidence from a cross-sectional study**

**Authors Information:**

Dr Ourania Kolokotroni, Department of Primary Care and Population Health, Medical School, University of Nicosia *

Dr Maria C Mosquera, Department of Primary Care and Population Health, Medical School, University of Nicosia *

Dr Annalisa Quattrocchi, Department of Primary Care and Population Health, Medical School, University of Nicosia

Dr Alexandros Heraclides, Department of Primary Care and Population Health, Medical School, University of Nicosia

Dr Christiana Demetriou, Department of Primary Care and Population Health, Medical School, University of Nicosia

Dr Elena Philippou, Department of Life and Health Sciences, School of Sciences and Engineering, University of Nicosia. Department of Nutritional Sciences, King’s College London.

*Equal contribution

Table 1. Adherence to Mediterranean Diet before and during Lockdown

|  |  | **The Month before Lockdown** | | **Month in Lockdown** | | **Score or Category change** | | **p-value^$^** |
| --- | --- | --- | --- | --- | --- | --- | --- | --- |
|  |  |  |  |  |  |  |  |  |
|  |  |  |  |  |  | **Decrease** | **Increase** |  |
| How many meals did you have daily? (including breakfast, lunch, dinner, morning, afternoon and evening snacks). | n (%) |  |  |  |  |  |  |  |
| 2 or less |  | 137 | 18.39 | 87 | 11.68 | 10.7 | 36.8 | <0.01* |
| 3 |  | 278 | 37.32 | 233 | 31.28 |  |  |  |
| 4 |  | 178 | 23.89 | 209 | 28.05 |  |  |  |
| 5 |  | 127 | 17.05 | 147 | 19.73 |  |  |  |
| 6 or more |  | 25 | 3.36 | 69 | 9.26 |  |  |  |
| Did you usually consume the main meals of the day (breakfast, lunch, dinner): | n (%) |  |  |  |  |  |  |  |
| Alone |  | 262 | 35.17 | 206 | 27.65 | 12.9 | 20.4 | <0.01 |
| With Friends and Family |  | 483 | 64.83 | 539 | 72.35 |  |  |  |
| Were you fasting during the month of quarantine? | n (%) |  |  |  |  |  |  |  |
| No |  |  |  | 559 | 75.03 |  |  |  |
| Yes |  |  |  | 186 | 24.97 |  |  |  |
| If so, please write the total number of days you fasted during the month in quarantine. | mean (SD) |  |  | 19.9 | 29.4 |  |  |  |
| Did you use olive oil as your main culinary fat? | n (%) |  |  |  |  |  |  |  |
| No |  | 108 | 14.5 | 97 | 13.02 | 1.1 | 2.6 | 0.05 |
| Yes |  | 637 | 85.5 | 648 | 86.98 |  |  |  |
| How many tablespoons of olive oil did you consume per day? (Including olive oil used for cooking, salads, out of house meals, etc). | n (%) |  |  |  |  |  |  |  |
| 1 or less |  | 254 | 34.09 | 225 | 30.2 | 3.8 | 12.5 | <0.01 |
| 2-3 |  | 396 | 53.15 | 387 | 51.95 |  |  |  |
| 4 or more |  | 95 | 12.75 | 133 | 17.85 |  |  |  |
| How many vegetable servings did you consume per day? (Including cooked and raw vegetables but not potatoes and beans). One serving = one large cup or half a large plate. | n (%) |  |  |  |  |  |  |  |
| Less than 1 |  | 105 | 14.09 | 103 | 13.83 | 12.5 | 18.1 | 0.02 |
| 1 |  | 277 | 37.18 | 251 | 33.69 |  |  |  |
| 2 |  | 237 | 31.81 | 248 | 33.29 |  |  |  |
| 3 or more |  | 126 | 16.91 | 143 | 19.19 |  |  |  |
| How many servings of fresh fruit did you consume per day? (One serving = one fruit unit of medium size, one large cup of sliced fruit, one slice of melon or watermelon of medium size, or one cup of freshly squeezed juice). | n (%) |  |  |  |  |  |  |  |
| Less than 1 |  | 145 | 19.46 | 121 | 16.24 | 13.4 | 26.3 | <0.01 |
| 1 |  | 285 | 38.26 | 244 | 32.75 |  |  |  |
| 2 |  | 234 | 31.41 | 261 | 35.03 |  |  |  |
| 3 or more |  | 81 | 10.87 | 119 | 15.97 |  |  |  |
| How many servings of red meat (pork, veal/beef, lamb), or red meat products (hamburgers, sausages, ham) did you consume per week? (One serving = 100 to 150 g = a quarter of a meal dish). | n (%) |  |  |  |  |  |  |  |
| 1 or less |  | 274 | 36.78 | 303 | 40.67 | 13.6 | 12.9 | <0.01 |
| 2-4 |  | 399 | 53.56 | 348 | 46.71 |  |  |  |
| 5-6 |  | 59 | 7.92 | 80 | 10.74 |  |  |  |
| 7 or more |  | 13 | 1.74 | 14 | 1.88 |  |  |  |
| How many servings of butter, margarine, or cream did you consume per day? (One serving = 12 g = one dessert spoon for butter and margarine; 2 tablespoons for cream). | n (%) |  |  |  |  |  |  |  |
| Less than 1 |  | 498 | 66.85 | 445 | 59.73 | 4.0 | 11.1 | <0.01 |
| 1 or more |  | 247 | 33.15 | 300 | 40.27 |  |  |  |
| How many sweet beverages did you consume per day? (e.g. carbonated orange or lemonade, cola, soda, etc., or sweet drinks such as chocolate, coffee with cream or whipped cream, standard cold tea with added sugar, liqueur or alcoholic beverages (except wine)). | n (%) |  |  |  |  |  |  |  |
| Less than 1 |  | 464 | 62.28 | 409 | 54.9 | 4.4 | 11.8 | <0.01 |
| 1 or more |  | 282 | 37.72 | 336 | 45.1 |  |  |  |
| How many glasses of wine did you consume per week? | n (%) |  |  |  |  |  |  |  |
| One or less (occasionally) |  | 582 | 78.12 | 586 | 78.66 | 7.4 | 8.6 | 0.08 |
| Two to six (sometimes but not daily) |  | 144 | 19.33 | 125 | 16.78 |  |  |  |
| Seven to fourteen (one or two glasses per day) |  | 14 | 1.88 | 24 | 3.22 |  |  |  |
| More than fourteen (more than two glasses per day) |  | 5 | 0.67 | 10 | 1.34 |  |  |  |
| How many servings of legumes did you consume per week? (beans, peas, chickpeas, lentils) One serving = 150 g = 2 cup. | n (%) |  |  |  |  |  |  |  |
| Less than 1 |  | 109 | 14.63 | 94 | 12.62 | 10.6 | 21.5 | <0.01 |
| 1 |  | 203 | 27.25 | 178 | 23.89 |  |  |  |
| 2 |  | 318 | 42.68 | 310 | 41.61 |  |  |  |
| 3 or more |  | 115 | 15.44 | 163 | 21.88 |  |  |  |
| How many servings of fish or shellfish did you consume per week? One serving = 100 to 150 g = a quarter of a meal dish. | n (%) |  |  |  |  |  |  |  |
| Less than 1 |  | 241 | 32.35 | 225 | 30.2 | 12.1 | 23.1 | <0.01 |
| 1 |  | 329 | 44.16 | 283 | 37.99 |  |  |  |
| 2 |  | 153 | 20.54 | 191 | 25.64 |  |  |  |
| 3 or more |  | 22 | 2.95 | 46 | 6.17 |  |  |  |
| How many times per week did you consume commercial sweets or pastries (not homemade), such as cakes, cookies, biscuits, or custard? | n (%) |  |  |  |  |  |  |  |
| Less than 1 |  | 273 | 36.64 | 285 | 38.26 | 17.9 | 28.1 | <0.01* |
| 1 |  | 194 | 26.04 | 142 | 19.06 |  |  |  |
| 2 |  | 137 | 18.39 | 132 | 17.72 |  |  |  |
| 3 |  | 79 | 10.6 | 78 | 10.47 |  |  |  |
| 4 or more |  | 62 | 8.32 | 108 | 14.5 |  |  |  |
| How many servings of nuts did you consume per week? One serving = 30 g = one handful (nuts include unsalted peanuts, almonds, hazelnuts, chestnut, walnuts). | n (%) |  |  |  |  |  |  |  |
| Less than 1 |  | 278 | 37.32 | 253 | 33.96 | 11.5 | 21.5 | <0.01 |
| 1 |  | 178 | 23.89 | 172 | 23.09 |  |  |  |
| 2 |  | 154 | 20.67 | 156 | 20.94 |  |  |  |
| 3 or more |  | 135 | 18.12 | 164 | 22.01 |  |  |  |
| Did you preferentially consume chicken, turkey or rabbit meat, or a vegetarian protein source, instead of red meat (veal/beef, pork, lamb) or any derived products (hamburgers, sausages, ham)? | n (%) |  |  |  |  |  |  |  |
| No, I prefer red meat instead of white |  | 150 | 20.13 | 156 | 20.94 | 5.1 | 4.3 | 0.55 |
| Yes, I prefer white meat or legumes ins |  | 595 | 79.87 | 589 | 79.06 |  |  |  |
| How many times per week did you consume dishes cooked with tomato or tomato sauce, onion and (or) garlic and olive oil? | n (%) |  |  |  |  |  |  |  |
| Less than 1 |  | 156 | 20.94 | 118 | 15.84 | 5.2 | 18.1 | <0.01 |
| 1 |  | 289 | 38.79 | 252 | 33.83 |  |  |  |
| 2 or more |  | 300 | 40.27 | 375 | 50.34 |  |  |  |
| How many servings of milk, yogurt or cheese did you consume per day? (One serving = 1 cup of milk, or 1 cup of yoghurt, or 2 thin slices of cheese). | n (%) |  |  |  |  |  |  |  |
| Less than 1 |  | 142 | 19.06 | 144 | 19.33 | 10.6 | 20.8 | <0.01 |
| 1 |  | 283 | 37.99 | 237 | 31.81 |  |  |  |
| 2 |  | 224 | 30.07 | 236 | 31.68 |  |  |  |
| 3 or more |  | 96 | 12.89 | 128 | 17.18 |  |  |  |
| Did you eat preferentially low-fat milk, yogurt, or cheese? | n (%) |  |  |  |  |  |  |  |
| No |  | 212 | 28.46 | 222 | 29.8 | 3.8 | 3.5 | <0.01 |
| Yes |  | 502 | 67.38 | 478 | 64.16 |  |  |  |
| Not applicable, as did not consume any dairy products |  | 31 | 4.16 | 45 | 6.04 |  |  |  |
| Did you eat preferentially whole cereals (non-refined cereals)? | n (%) |  |  |  |  |  |  |  |
| No |  | 280 | 37.58 | 297 | 39.87 | 4.4 | 2.1 | 0.02 |
| Yes |  | 465 | 62.42 | 448 | 60.13 |  |  |  |
| How many cups of caffeinated drinks did you have per day? (Including coffee, black or green tea, cola drinks, or energy drinks). | n (%) |  |  |  |  |  |  |  |
| Less than 1 |  | 150 | 20.13 | 163 | 21.88 | 8.9 | 10.9 | <0.01 |
| 1 - 3 |  | 479 | 64.3 | 438 | 58.79 |  |  |  |
| > 3 |  | 116 | 15.57 | 144 | 19.33 |  |  |  |
| How many cups of infusions/herbal teas or relaxing drinks did you have per day? | n (%) |  |  |  |  |  |  |  |
| Less than 1 |  | 489 | 65.64 | 479 | 64.3 | 6.7 | 9.9 | <0.01 |
| 1 - 3 |  | 229 | 30.74 | 218 | 29.26 |  |  |  |
| > 3 |  | 27 | 3.62 | 48 | 6.44 |  |  |  |
| How much water did you drink per day? | n (%) |  |  |  |  |  |  |  |
| Less than one liter (4 glasses) |  | 264 | 35.44 | 256 | 34.36 | 12.6 | 14.6 | 0.28 |
| One to two liters (4 to eight glasses) |  | 356 | 47.79 | 362 | 48.59 |  |  |  |
| More than two liters (more than eight glasses) |  | 125 | 16.78 | 127 | 17.05 |  |  |  |
| **Mediterrranean Diet Score** | median (IQR) | 6 | 2 | 6 | 3 | 22.7 | 31.9 | <0.01 |

^$^Exact test for symmetry p-values for categorical variables, and Signed rank test p-values for continuous variables

*Exact test for symmetry not feasible, reporting Symmetry (asymptotic) p-value

Table 2. Physical Activity levels before and during Lockdown

|  |  | **The Month before Lockdown** | | **Lockdown** | | **Score or Category change** | | **p-value*** |
| --- | --- | --- | --- | --- | --- | --- | --- | --- |
|  |  |  |  |  |  | **Decrease** | **Increase** |  |
| **PHYSICAL ACTIVITY** | | | | | | | | |
| **MET walking** | median (IQR) | 231 | 528 | 297 | 643.5 | 25.8 | 37.9 | <0.01 |
| **MET moderate** | median (IQR) | 180 | 480 | 120 | 480 | 20.1 | 22.0 | 0.60 |
| **MET vigorous** | median (IQR) | 600 | 1440 | 360 | 1440 | 24.6 | 21.6 | 0.13 |
| **MET total** | median (IQR) | 792 | 1880 | 813 | 1815 | 37.0 | 43.8 | 0.16 |
| **Time spent sitting (mins)** | median (IQR) | 120 | 340 | 180 | 466 | 14.5 | 67.1 | <0.01 |
| **PHYSICAL ACTIVITY SCORE** | n (%) |  |  |  |  |  |  |  |
| Low |  | 368 | 49.4 | 364 | 48.86 | 21.2 | 21.5 | 0.95 |
| Moderate |  | 188 | 25.23 | 196 | 26.31 |  |  |  |
| High |  | 189 | 25.37 | 185 | 24.83 |  |  |  |

*Signrank test was used for the comparison of median questionnaire scores between the months of February 2020 (i.e. - the Month before Lockdown) and Lockdown and Bowker symmetry Test for comparison of categorical variables

Table 3. Stress levels before and during Lockdown

|  |  | **The Month before Lockdown** | | **Month in Lockdown** | | **Score or Category change** | | **p-value*** |
| --- | --- | --- | --- | --- | --- | --- | --- | --- |
|  |  |  |  |  |  |  |  |  |
|  |  |  |  |  |  | **Decrease** | **Increase** |  |
| How often were you upset because of something that happened unexpectedly? | median (IQR) | 2 | 1 | 2 | 2 | 12.3 | 37.3 | <0.01 |
| How often did you feel that you were unable to control the important things in your life? | median (IQR) | 2 | 1 | 2 | 2 | 8.5 | 38.9 | <0.01 |
| How often did you feel nervous and stressed? | median (IQR) | 2 | 1 | 2 | 1 | 17.3 | 36.6 | <0.01 |
| How often did you deal successfully with day to day problems and annoyances? | median (IQR) | 3 | 2 | 3 | 2 | 21.9 | 8.3 | <0.01 |
| How often did you feel that you were effectively coping with important changes that were occurring in your life? | median (IQR) | 3 | 2 | 3 | 1 | 23.5 | 9.7 | <0.01 |
| How often did you feel confident about your ability to handle your personal problems? | median (IQR) | 3 | 2 | 3 | 2 | 24.6 | 7.5 | <0.01 |
| How often did you feel that things were going your way? | median (IQR) | 3 | 1 | 2 | 1 | 38.9 | 7.4 | <0.01 |
| How often did you find that you could not cope with all the things that you had to do? | median (IQR) | 2 | 1 | 2 | 1 | 16.9 | 27.5 | <0.01 |
| How often were you able to control irritations in your life? | median (IQR) | 3 | 1 | 3 | 1 | 28.7 | 9.1 | <0.01 |
| How often did you feel that you were on top of things? | median (IQR) | 3 | 1 | 2 | 1 | 33.8 | 7.4 | <0.01 |
| How often were you angered because of things that happened that were outside of your control? | median (IQR) | 2 | 2 | 2 | 1 | 12.3 | 30.9 | <0.01 |
| How often did you find yourself thinking about things that you had to accomplish? | median (IQR) | 3 | 2 | 3 | 2 | 16.6 | 20.1 | 0.17 |
| How often were you able to control the way you spent your time? | median (IQR) | 3 | 2 | 3 | 1 | 29.4 | 17.9 | <0.01 |
| How often did you feel difficulties were piling up so high that you could not overcome them? | median (IQR) | 2 | 1 | 2 | 1 | 12.8 | 26.0 | <0.01 |
| **PSS-14 overall score** | median (IQR) | 22 | 9 | 25 | 12 | 21.7 | 57.9 | <0.01 |

*Signrank test for the comparison of median values

Table 4. Sleep before and during Lockdown

|  |  | **The Month before Lockdown** | | **Month in Lockdown** | | **Score or Category change** | | **p-value*** |
| --- | --- | --- | --- | --- | --- | --- | --- | --- |
|  |  |  |  |  |  |  |  |  |
|  |  |  |  |  |  | **Decrease** | **Increase** |  |
| **Sleep duration** | median (IQR) | 0 | 1 | 0 | 1 | 20.5 | 15.7 | 0.08 |
| **Total hours of sleep** | median (IQR) | 7.75 | 1.5 | 8 | 2.75 | 29.6 | 42.6 | <0.01 |
| **Sleep disturbances** | median (IQR) | 1 | 0 | 1 | 0 | 4.7 | 19.3 | <0.01 |
| **How often did you have trouble sleeping because you could not get to sleep within 30 minutes?** | n (%) |  |  |  |  |  |  |  |
| Not during that month |  | 333 | 44.7 | 256 | 34.36 | 6.6 | 39.7 | <0.01 |
| Less than once a week |  | 246 | 33.02 | 152 | 20.4 |  |  |  |
| Once or twice a week |  | 118 | 15.84 | 160 | 21.48 |  |  |  |
| Three or more times a week |  | 48 | 6.44 | 177 | 23.76 |  |  |  |
| **How often did you have trouble sleeping because you woke up in the middle of the night or early morning?** | n (%) |  |  |  |  |  |  |  |
| Not during that month |  | 374 | 50.2 | 289 | 38.79 | 9.1 | 33.3 | <0.01 |
| Less than once a week |  | 206 | 27.65 | 164 | 22.01 |  |  |  |
| Once or twice a week |  | 104 | 13.96 | 159 | 21.34 |  |  |  |
| Three or more times a week |  | 61 | 8.19 | 133 | 17.85 |  |  |  |
| **How often did you have trouble sleeping because you had to get up to use the bathroom?** | n (%) |  |  |  |  |  |  |  |
| Not during that month |  | 388 | 52.08 | 351 | 47.11 | 3.5 | 16.1 | <0.01 |
| Less than once a week |  | 183 | 24.56 | 159 | 21.34 |  |  |  |
| Once or twice a week |  | 96 | 12.89 | 118 | 15.84 |  |  |  |
| Three or more times a week |  | 78 | 10.47 | 117 | 15.7 |  |  |  |
| **How often did you have trouble sleeping because you could not breathe comfortably?** | n (%) |  |  |  |  |  |  |  |
| Not during that month |  | 634 | 85.1 | 591 | 79.33 | 3.5 | 12.3 | <0.01 |
| Less than once a week |  | 74 | 9.93 | 82 | 11.01 |  |  |  |
| Once or twice a week |  | 28 | 3.76 | 46 | 6.17 |  |  |  |
| Three or more times a week |  | 9 | 1.21 | 26 | 3.49 |  |  |  |
| **How often did you have trouble sleeping because you coughed or snored loudly?** | n (%) |  |  |  |  |  |  |  |
| Not during that month |  | 630 | 84.56 | 633 | 84.97 | 5.2 | 6.3 | 0.31 |
| Less than once a week |  | 77 | 10.34 | 63 | 8.46 |  |  |  |
| Once or twice a week |  | 28 | 3.76 | 40 | 5.37 |  |  |  |
| Three or more times a week |  | 10 | 1.34 | 9 | 1.21 |  |  |  |
| **How often did you have trouble sleeping because you felt too cold?** | n (%) |  |  |  |  |  |  |  |
| Not during that month |  | 600 | 80.54 | 624 | 83.76 | 9.0 | 5.2 | 0.03 |
| Less than once a week |  | 101 | 13.56 | 84 | 11.28 |  |  |  |
| Once or twice a week |  | 35 | 4.7 | 27 | 3.62 |  |  |  |
| Three or more times a week |  | 9 | 1.21 | 10 | 1.34 |  |  |  |
| **How often did you have trouble sleeping because you felt too hot?** | n (%) |  |  |  |  |  |  |  |
| Not during that month |  | 606 | 81.34 | 472 | 63.36 | 3.6 | 25.6 | <0.01 |
| Less than once a week |  | 94 | 12.62 | 148 | 19.87 |  |  |  |
| Once or twice a week |  | 31 | 4.16 | 94 | 12.62 |  |  |  |
| Three or more times a week |  | 14 | 1.88 | 31 | 4.16 |  |  |  |
| **How often did you have trouble sleeping because you had bad dreams?** | n (%) |  |  |  |  |  |  |  |
| Not during that month |  | 509 | 68.32 | 427 | 57.32 | 6.4 | 23.9 | <0.01 |
| Less than once a week |  | 174 | 23.36 | 182 | 24.43 |  |  |  |
| Once or twice a week |  | 51 | 6.85 | 95 | 12.75 |  |  |  |
| Three or more times a week |  | 11 | 1.48 | 41 | 5.5 |  |  |  |
| **How often did you have trouble sleeping because you had pain?** | n (%) |  |  |  |  |  |  |  |
| Not during that month |  | 541 | 72.62 | 504 | 67.65 | 5.2 | 16.1 | <0.01 |
| Less than once a week |  | 134 | 17.99 | 121 | 16.24 |  |  |  |
| Once or twice a week |  | 45 | 6.04 | 71 | 9.53 |  |  |  |
| Three or more times a week |  | 25 | 3.36 | 49 | 6.58 |  |  |  |
| **How often did you have trouble sleeping because of other reason(s)? Please describe, including how often you had trouble sleeping because of this reason(s):** | n (%) |  |  |  |  |  |  |  |
| Not during that month |  | 688 | 92.35 | 677 | 90.87 | 1.2 | 5.5 | <0.01 |
| Less than once a week |  | 19 | 2.55 | 14 | 1.88 |  |  |  |
| Once or twice a week |  | 21 | 2.82 | 17 | 2.28 |  |  |  |
| Two to three times a week |  | 4 | 0.54 | 3 | 0.4 |  |  |  |
| Three or more times a week |  | 13 | 1.74 | 34 | 4.56 |  |  |  |
| **Sleep latency** | median (IQR) | 1 | 1 | 1 | 2 | 6.1 | 40.6 | <0.01 |
| **Daytime dysfunction** | median (IQR) | 1 | 1 | 1 | 1 | 12.3 | 28.5 | <0.01 |
| **Sleep efficiency** | median (IQR) | 0 | 1 | 0 | 0 | 15.6 | 14.1 | 0.56 |
| **Sleep medication** | median (IQR) | 0 | 0 | 0 | 0 | 1.2 | 4.3 | <0.01 |
| **Sleep quality** | median (IQR) | 1 | 1 | 1 | 2 | 10.1 | 26.8 | <0.01 |
| **PSQI score** | median (IQR) | 4 | 4 | 5 | 4 | 25.3 | 49.1 | <0.01 |

* Symmetry exact p-value for categorical variables and Signrank test for comparison of medians

Table 5. Social Connections before and during Lockdown

|  |  | **The Month**  **before Lockdown** | | **Month in Lockdown** | | **Score or  Category change** | | **p-value*** |
| --- | --- | --- | --- | --- | --- | --- | --- | --- |
|  |  |  |  |  |  |  |  |  |
|  |  |  |  |  |  | **Decrease** | **Increase** |  |
| **Emotional / Informational Support** | **median (IQR)** | **71.875** | **37.5** | **65.625** | **40.625** | **28.9** | **12.5** | **<0.01** |
| Someone you can count on to listen to you | median (IQR) | 5 | 1 | 5 | 1 | 13.4 | 5.5 | <0.01 |
| Someone to give you information | median (IQR) | 5 | 1 | 5 | 1 | 15.2 | 3.6 | <0.01 |
| Someone to give you advice about a crisis | median (IQR) | 5 | 1 | 5 | 1 | 16.1 | 4.7 | <0.01 |
| Someone to confide in or talk about your problems | median (IQR) | 5 | 1 | 5 | 1 | 13.0 | 4.3 | <0.01 |
| Someone whose advice you really want | median (IQR) | 5 | 1 | 5 | 1 | 14.8 | 4.6 | <0.01 |
| Someone to share your most private worries and fears | median (IQR) | 5 | 1 | 5 | 1 | 13.6 | 5.2 | <0.01 |
| Someone to turn to for suggestions to deal with problems | median (IQR) | 5 | 1 | 5 | 1 | 12.6 | 4.3 | <0.01 |
| Someone who understands your problems | median (IQR) | 5 | 1 | 5 | 1 | 12.9 | 4.4 | <0.01 |
| **Tangible Support** | **median (IQR)** | **75** | **43.75** | **75** | **43.75** | **20.7** | **13.4** | **0.0007** |
| Someone to help you if you were confined to bed | median (IQR) | 5 | 1 | 5 | 1 | 11.0 | 5.1 | <0.01 |
| Someone to take you to the doctor if you needed it | median (IQR) | 5 | 1 | 5 | 1 | 9.8 | 4.0 | <0.01 |
| Someone to prepare your meals if you were unable | median (IQR) | 5 | 1 | 5 | 1 | 9.9 | 8.1 | 0.2446 |
| Someone to help with daily chores if you were sick | median (IQR) | 5 | 1 | 5 | 1 | 11.3 | 7.2 | 0.0103 |
| **Affectionate Support** | **median (IQR)** | **75** | **50** | **75** | **50** | **24.6** | **9.7** | **<0.01** |
| Someone who shows you love and affection | median (IQR) | 5 | 1 | 5 | 1 | 9.3 | 3.9 | <0.01 |
| Someone to love and make you feel wanted | median (IQR) | 5 | 1 | 5 | 1 | 10.1 | 4.4 | <0.01 |
| Someone who hugs you | median (IQR) | 5 | 1 | 5 | 2 | 19.3 | 4.7 | <0.01 |
| **Positive Social Interaction** | **median (IQR)** | **75** | **50** | **66.67** | **58.333** | **34** | **11** | **<0.01** |
| Someone to have a good time with | median (IQR) | 5 | 1 | 5 | 1 | 22.6 | 5.9 | <0.01 |
| Someone to get together with for relaxation | median (IQR) | 5 | 1 | 5 | 2 | 24.3 | 6.6 | <0.01 |
| Someone to do something enjoyable with | median (IQR) | 5 | 1 | 4 | 2 | 28.1 | 5.6 | <0.01 |
| **Overall Support Index** | **median (IQR)** | **71.05** | **31.58** | **68.42** | **36.84** | **43.6** | **18.0** | **<0.01** |

*Signrank test for the comparison of median values
